# The circEPSTI1/mir-942-5p/LTBP2 axis regulates the progression of OSCC in the background of OSF via EMT and the PI3K/Akt/mTOR pathway

**DOI:** 10.1038/s41419-020-02851-w

**Published:** 2020-08-12

**Authors:** Jie Wang, Canhua Jiang, Ning Li, Fei Wang, Ying Xu, Zhengzhong Shen, Lina Yang, Zaiye Li, Caiyun He

**Affiliations:** 1 Department of Oral and Maxillofacial Surgery, Center of Stomatology, Xiangya Hospital, Central South University, 410008 Changsha, China; 2 Department of Immunology, School of Basic Medical Science, Central South University, 410008 Changsha, China; 3 Institute of Oral Precancerous Lesions, Central South University, 410008 Changsha, China; 4 Research Center of Oral and Maxillofacial Tumor, Xiangya Hospital, Central South University, 410008 Changsha, China

**Keywords:** Oral cancer, Prognostic markers

## Abstract

Oral squamous cell carcinoma (OSCC) in the background of oral submucous fibrosis (OSF) caused by areca nut chewing has a high incidence in Asia-Pacific countries. However, the molecular mechanism remains unclear. Here, we performed circRNA microarray analysis to screen the circRNA expression profiles in OSCC and OSF. We identified circEPSTI1 as a circRNA with consistent, sequential upregulation from normal buccal mucosa (NBM) to OSF to OSCC. Functionally, circEPSTI1 significantly promoted OSCC cell proliferation and invasion, as evidenced by the CCK8, colony formation, wound healing, and transwell assays with circEPSTI1 overexpression and silencing. OSCC patients with circEPSTI1^high^ status exhibited poor prognoses. CircEPSTI1 sponged miR-942-5p and accelerated epithelial-mesenchymal transition (EMT) to increase LTBP2 expression in OSCC through phosphorylation of PI3K/Akt/mTOR signaling pathway components. Blocking the PI3K/Akt/mTOR signaling pathway with the dual PI3k/mTOR inhibitor BEZ235 reversed OSCC progression induced by overexpression of circEPSTI1 and LTBP2. Collectively, these results indicate that the circEPSTI1/miR-942-5p/LTBP2 axis affects OSCC cell proliferation and invasion via the acceleration of EMT and the phosphorylation of PI3K/Akt/mTOR signaling pathway components. CircEPSTI1 may be an independent diagnostic and prognostic marker and a potential therapeutic target for OSCC patients with OSF.

## Introduction

Tobacco or alcohol use, betel quid or areca nut chewing, sharp residual roots and crowns of teeth and human papillomavirus are well-established etiologic agents for oral squamous cell carcinoma (OSCC). Among these agents, areca nut, which is commonly chewed in South and Southeast Asian countries and some provinces in China, is a highly unique pathogenic factor. First, areca nut is an independent group I human carcinogen according to World Health Organization monographs^1^. Second, areca nut chewing can result in oral submucous fibrosis (OSF), a chronic and insidious oral precancerous condition associated with a 3–6% risk of OSCC^2^. The inflammatory reaction in the juxta-epithelial region and subsequent excessive collagen deposition in the underlying submucosal layer are the characteristic histopathologic features of OSF^3^. Moreover, the major clinical symptom of OSF patients is trismus, which limits the ability of patients to open the mouth and eventually impairs eating, speaking, and dental care^4^.

OSCC in the background of OSF is one of the most common malignancies in South and Southeast Asian countries and China because of the increasing popularity of commercial areca nut and the increased uptake by young people^5^. In past decades, substantial efforts and advances have been made to improve OSCC treatment. However, the prognosis of OSCC patients is not yet optimistic^6^. Moreover, OSCC in the background of OSF has clinicopathological and prognostic features different from those of OSCC without OSF. Many reports from India indicate that better tumor differentiation, slower growth, a lower probability of cervical metastasis, a lower probability of postoperative recurrence, more favorable prognosis and higher survival rates are distinctive features of OSCC with OSF^7,8^. However, Guo et al. and Jun et al. found that OSCC patients with OSF in China have less favorable clinicopathological features and prognoses than OSCC patients without OSF^9,10^. These completely opposite results, on the one hand, could be explained by the possibility that differences in the betel quid and areca nut processing methods between the South/Southeast Asian countries and China could result in different immune responses in the oral mucosa. On the other hand, the actual molecular mechanism of OSF and its cancerization remain unclear, although the immune and inflammatory responses in submucosal cells may be the main cause^7^. OSCC with OSF is currently a public health problem in China, South Asian and Southeast Asian countries, and even some Western countries. Because of these serious challenges, identifying novel diagnostic and therapeutic biomarkers for OSCC in the background of OSF is an urgent need.

A class of noncoding RNAs, including circular RNAs (circRNAs) account for more than 90% of the transcriptome in humans but have little protein-coding potential^11,12^. CircRNAs are more stable and resistant to the degradation machinery than linear RNAs because they are derived from back spliced precursor mRNAs and covalently closed transcripts^13,14^. CircRNAs are cell-, tissue- or development-specific and can mediate cellular biological processes via diverse mechanisms, however, the process of their biogenesis and their potential function are poorly understood^15^. With the development of bioinformatics and RNA-seq technology in recent decade, circRNAs have attracted much attention in various cancer studies as microRNA (miRNA) sponges, RNA-binding protein sponges, and regulators of gene transcription and expression^16,17^. Several circRNAs have been reported to regulate OSCC tumorigenesis. For example, circ_0001971 was found to regulate OSCC progression and chemosensitivity by targeting miR-194/miR-204^18^. CircRNA_0000140 can suppress SCC growth and metastasis in the head and neck region by targeting the miR-31/Hippo axis^19^. However, no reports have described the role of circRNAs in OSCC in the background of OSF, which is the most common type of OSCC in China, South Asian countries, and Southeast Asian countries.

In this study, we used high-throughput RNA-seq technology to analyze the circRNA expression profiles in OSCC with OSF. We found for the first time that the significantly upregulated circEPSTI1 could be a prognostic marker for survival in OSCC patients and can promote OSCC cell proliferation and invasion by binding to miR-942-5p as a miRNA sponge to regulate latent transforming growth factor beta binding protein 2 (LTBP2) expression. Moreover, blocking the PI3K/Akt/mTOR signaling pathway can reverse OSCC cell proliferation and invasion induced by the circEPSTI1/miR-942-5p/LTBP2 axis.

## Materials and methods

### Patients and specimens

From May 2015 to January 2018, a total of 162 paired biopsies of OSCC and OSF tissue from 162 primary OSCC patients with OSF were collected at Xiangya Hospital. Moreover, 38 unmatched NBM tissues were procured from healthy volunteers who did not have the habit of areca nut chewing. Each specimen was divided into at least two parts: one part was fixed with formalin and embedded in paraffin for pathologic confirmation and immunohistochemical (IHC) staining, while the other was immediately stored in liquid nitrogen for further assays. All enrolled patients signed informed consent forms prior to treatment. The detailed clinicopathological data are shown in Supplemental Materials S3. The experimental protocols were approved by the Ethics Committee of Xiangya Hospital and conducted in accordance with the Declaration of Helsinki. The following patients were excluded: (1) patients <20 or >65 years old or without full civil capacity; (2) patients with a history of preoperative radiotherapy or chemotherapy or of treatment with biological agents or traditional Chinese medicines; (3) patients with incomplete postoperative follow-up data; and (4) patients with a history of another organ malignancy or systemic immune disease. All patients were followed up periodically. If a patient died or recurrence was found, the survival data of that patient were censored. Overall survival (OS) and progression-free survival (PFS) data were recorded.

### Cell culture

Four human OSCC cell lines (CAL27, HN6, UM1, and SCC9) and normal HOKs were purchased from the American Type Culture Collection (ATCC) (Manassas, VA, USA). The 293 T cell line was purchased from the Shanghai Institute of Cell Biology, Chinese Academy of Sciences (Shanghai, China). Details are provided in Supplemental Methods.

### CircRNA microarray analysis

Three paired OSCC and OSF tissues, as well as three NBM tissues, was investigated by circRNA microarray analysis. Details are provided in Supplemental Methods.

Quantitative real-time polymerase chain reaction (RT- qPCR), western blot, IHC staining, fluorescence in situ hybridization (FISH), circRNA precipitation, RNA immunoprecipitation (RIP), luciferase reporter, 5-ethynyl-2′-deoxyuridine (EdU) assays, cell proliferation, colony formation, wound healing cell migration, and Matrigel invasion assays

Details of the above assays are provided in Supplemental Methods. The RT-qPCR primers used in this study are listed in Supplemental Sequences. The antibodies used in this study are listed in Supplemental Antibodies.

### Oligonucleotide transfection

Cells were transfected using Lipofectamine 2000 (Invitrogen Life Technologies, Carlsbad, CA, USA). All circRNA siRNAs, and miRNA mimics and inhibitors were synthesized by GeneCopoeia (Rockville, MD, USA). The sequences used are presented in Supplemental Sequences. Details are provided in Supplemental Methods.

### Mouse xenograft model

Ethical approval was obtained from the Institute Research Ethics Committee of Xiangya hospital. Details are provided in Supplemental Methods.

### Statistical analysis

All statistical analyses were performed using the SPSS 22.0 software package (SPSS, Chicago, IL, USA). Quantitative data are presented as the means ± standard deviations (SDs). The Chi-squared test was used to investigate the significance of the correlation of circEPSTI1 expression with clinicopathological features in OSCC. Survival curves were constructed by the Kaplan–Meier method and compared with the log-rank test. Survival was measured from the day of surgery. *P* < 0.05 was considered statistically significant.

## Results

### CircEPSTI1 expression is consistently upregulated from normal buccal mucosa (NBM) to OSF to OSCC

**Fig. 1:**
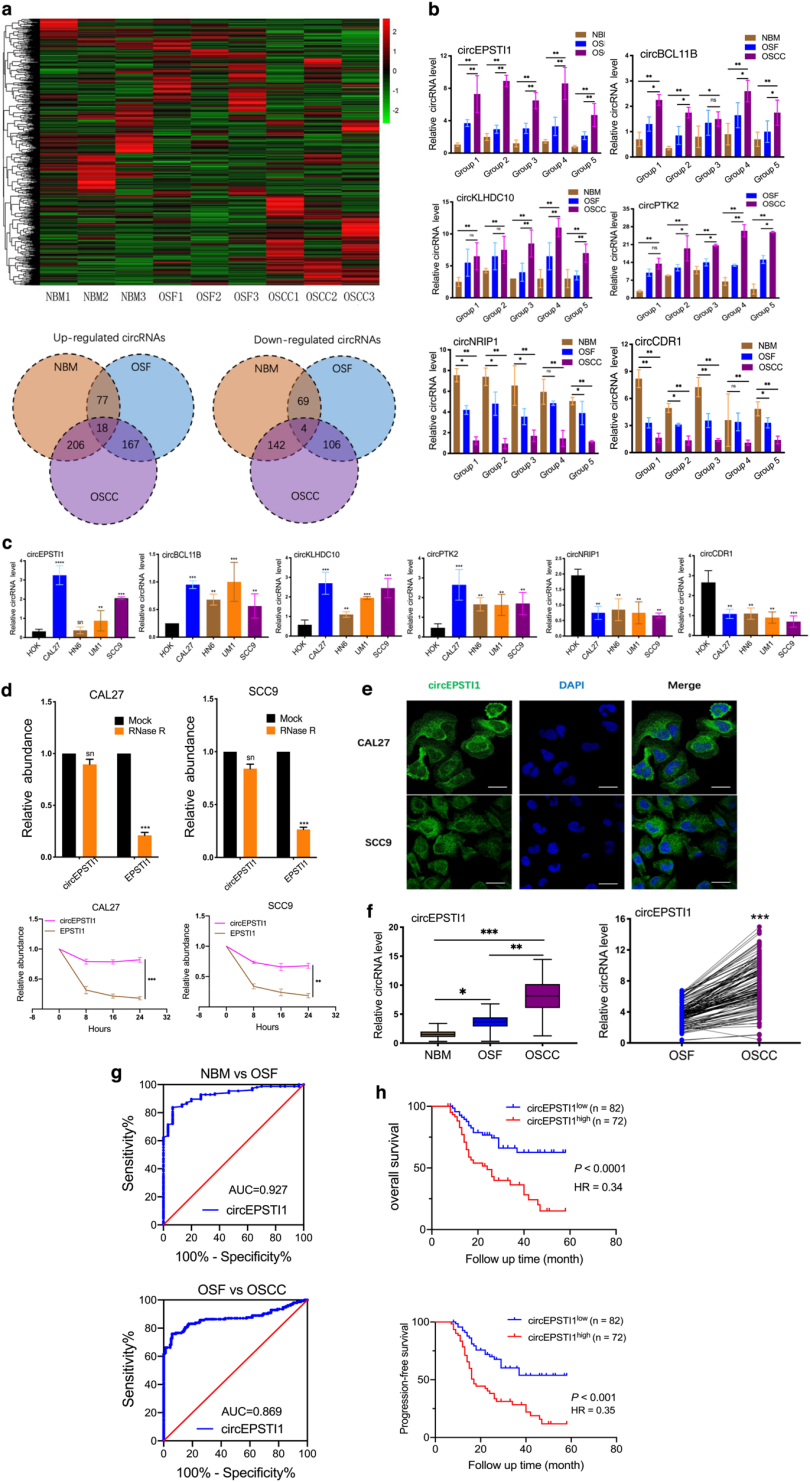
The expression of circEPSTI1 is consistently upregulated sequentially from NBM to OSF to OSCC. **a** Expression profile of circRNAs in three paired OSCC and matched OSF tissues as well as in three NBM tissues. **b, c** Six consistently deregulated circRNAs in tissue samples (**b**) and OSCC cell lines (**c**). **d** RNase R exonuclease and actinomycin D assays confirmed the circular structure of circEPSTI1. **e** The subcellular distribution of circEPSTI1 was cytoplasmic, as shown by FISH (scale bar 10 μm). **f** The expression level of circEPSTI1 in 154 OSCC and matched OSF tissues was analyzed by RT-qPCR. **g** ROC curve analysis revealed that circEPSTI1 could sensitively discriminate OSCC from OSF and OSF from NBM. **h** Kaplan–Meier analysis of the correlation between circEPSTI1 expression and OS as well as PFS. ***P < 0.001; **P < 0.01; *P < 0.05.

The expression profile of circRNAs in three paired OSCC and OSF tissues, as well as three NBM tissues, was investigated by circRNA microarray analysis. As shown in Fig. 1a, the variations in circRNA expression are revealed in the hierarchical clustering and Venn-diagram. A total of 95 circRNAs were significantly upregulated, and 73 were downregulated in OSF tissue compared with NBM (Supplemental Materials S1, OSF VS NBM). Moreover, 295 circRNAs were differentially expressed between OSCC and OSF tissue, namely, 185 upregulated circRNAs and 110 downregulated circRNAs in OSCC tissue (Supplemental Materials S1, OSCC VS OSF). Most importantly, 22 circRNAs with a consistent sequential change from NBM to OSF to OSCC were identified (Supplemental Materials S1, OSCC VS OSF VS NBM), among which 18 were consistently upregulated and four were consistently downregulated. Four upregulated circRNAs [hsa_circ_0000479 (hsa_circ_EPSTI1), hsa_circ_0002162 (hsa_circ_PTK2), hsa_circ_0002190 (hsa_circ_KLHDC10), and hsa_circ_0033144 (hsa_circ_BCL11B)] and two downregulated circRNAs [hsa_circ_0004771 (hsa_circ_NRIP1) and hsa_circ_0001946 (hsa_circ_CDR1)] were validated in five groups of NBM, OSF, and OSCC specimens and four OSCC cell lines (CAL27, HN6, UM1, and SCC9) with human oral keratinocytes (HOKs) as normal control cells by RT- qPCR. The expression pattern of each validated circRNA was consistent with the microarray data. However, notably, circEPSTI1, which is located on chromosome 13q14 (chr13:43528083–43544806) and confirmed by Sanger sequencing (Supplemental Fig. Sa), exhibited the most significant upregulation in OSCC tissue compared to OSF (mean, 2.4-fold) and NBM (mean, 5.5-fold) tissue (Fig. 1b). The strongest upregulation of circEPSTI1 was found in CAL27 cells, followed by SCC9 cells (Fig. 1c). In addition, as shown in Fig. 1d, resistance to digestion by RNase R exonuclease further confirmed that circEPSTI1 is circular. The results of actinomycin D assays revealed that the half-life of the circEPSTI1 transcript exceeded 24 h, longer than the 4-h half-life of EPSTI1 mRNA, indicating that circEPSTI1 is more stable than the linear EPSTI1 transcript. Hence, circEPSTI1 was selected for the evaluation of OSCC tumorigenesis and proliferation in the present study.

**Table 1:** Association between circEPSTI1, miR-942-5p, and LTBP2 expressions and clinical parameters in 154 OSCCs arising in the background of OSF.

Item	Cases (%)	circEPSTI1 level				miR-942-5p level				LTBP2 level		
		Low	High	*P*	Low		High	*P*	Low		High	*P*
Gender												
Male	129 (83.8)	69	60	0.891	62		67	0.718	55		74	0.807
Female	25 (16.2)	13	12		13		12		10		15	
Age												
≤40 yr	47 (30.5)	21	26	0.089	22		25	0.755	17		30	0.314
>40 yr	107 (69.5)	61	46		53		54		48		59	
T stage												
T1/T2	81 (52.5)	53	28	0.001^*^	33		48	0.037^a^	42		39	0.010^*^
T3/T4	73 (47.5)	29	44		42		31		23		50	
N stage												
N0	86 (55.8)	51	35	0.092	36		50	0.080	41		45	0.122
N1/N2	68 (44.2)	31	37		39		29		24		44	
TNM stage												
I/II	71 (46.1)	47	24	0.002^*^	26		45	0.005^a^	37		34	0.021^*^
III/IV	83 (53.9)	35	48		49		34		28		55	
OSCC diff.												
Well	102 (66.2)	59	43	0.340	51		51	0.662	45		57	0.896
Moderated	35 (22.7)	17	18	0.365	16		19	0.927	15		20	0.349
Poor	17 (11.1)	6	11		8		9		5		12	
OSF stage												
Early	35 (22.7)	18	17	0.926	17		18	0.762	10		25	0.066
Moderated	97 (62.9)	49	48	0.133	50		47	0.198	45		52	0.936
Advanced	22 (14.4)	15	7		8		14		10		12	

*P < 0.05.

**Table 2:** Association between circEPSTI1/miR-942-5p/LTBP2 expression and clinicopathological parameters in 101 OSCCs arising in the background of OSF.

Item	Cases (%)	circEPSTI1/miR-942-5p/LTBP2		
		circEPSTI1^high^/miR-942-5p^low^/LTBP2^high^	circEPSTI1^low^/miR-942-5p^high^/LTBP2^low^	*P*
Gender				
Male	84 (83.2)	45	39	0.962
Female	17 (16.8)	9	8	
Age				
≤40 yr	28 (27.7)	16	12	0.560
>40 yr	73 (72.3)	37	36	
T stage				
T1/T2	53 (52.5)	21	32	0.006^*^
T3/T4	48 (47.5)	32	16	
N stage				
N0	57 (56.4)	25	32	0.076
N1/N2	44 (43.6)	28	16	
TNM stage				
I/II	46 (45.5)	17	29	0.004^*^
III/IV	55 (54.5)	36	19	
OSCC diff.				
Well	68 (67.3)	33	35	0.623
Moderated	22 (21.8)	12	10	0.618
Poor	11 (10.9)	7	4	
OSF stage				
Early	20 (19.8)	12	8	0.583
Moderated	66 (65.3)	35	31	0.362
Advanced	15 (14.9)	6	9	

*P < 0.05.

We found that circEPSTI1 was distributed predominantly in the cytoplasm of OSCC cells via cellular FISH (Fig. 1e). Furthermore, RT-qPCR on 154 paired samples of OSCC and OSF tissues showed that the expression of circEPSTI1 was sequentially and significantly increased from NBM to OSF to OSCC (Fig. 1f). Receiver operating characteristic (ROC) curve revealed that circEPSTI1 could sensitively discriminate OSCC from OSF and OSF from NBM (Fig. 1g). As shown in Table 1, high expression of circEPSTI1 (circEPSTI1 expression ratio ≥ median ratio) was significantly correlated with high T stage (*P* < 0.01) and advanced TNM stage (*P* < 0.01) in OSCC patients. But there is no significant correlation between expression of circEPSTI1 and N stage classification as well as OSF stage classification (*P* > 0.05). Kaplan–Meier survival analysis showed that higher expression of circEPSTI1 was significantly correlated with poorer OS and PFS (Fig. 1h). Hence, circEPSTI1 expression could serve as a prognostic biomarker for OSCC with OSF.

### CircEPSTI1 overexpression promotes malignant behaviors of OSCC cells

**Fig. 2:**
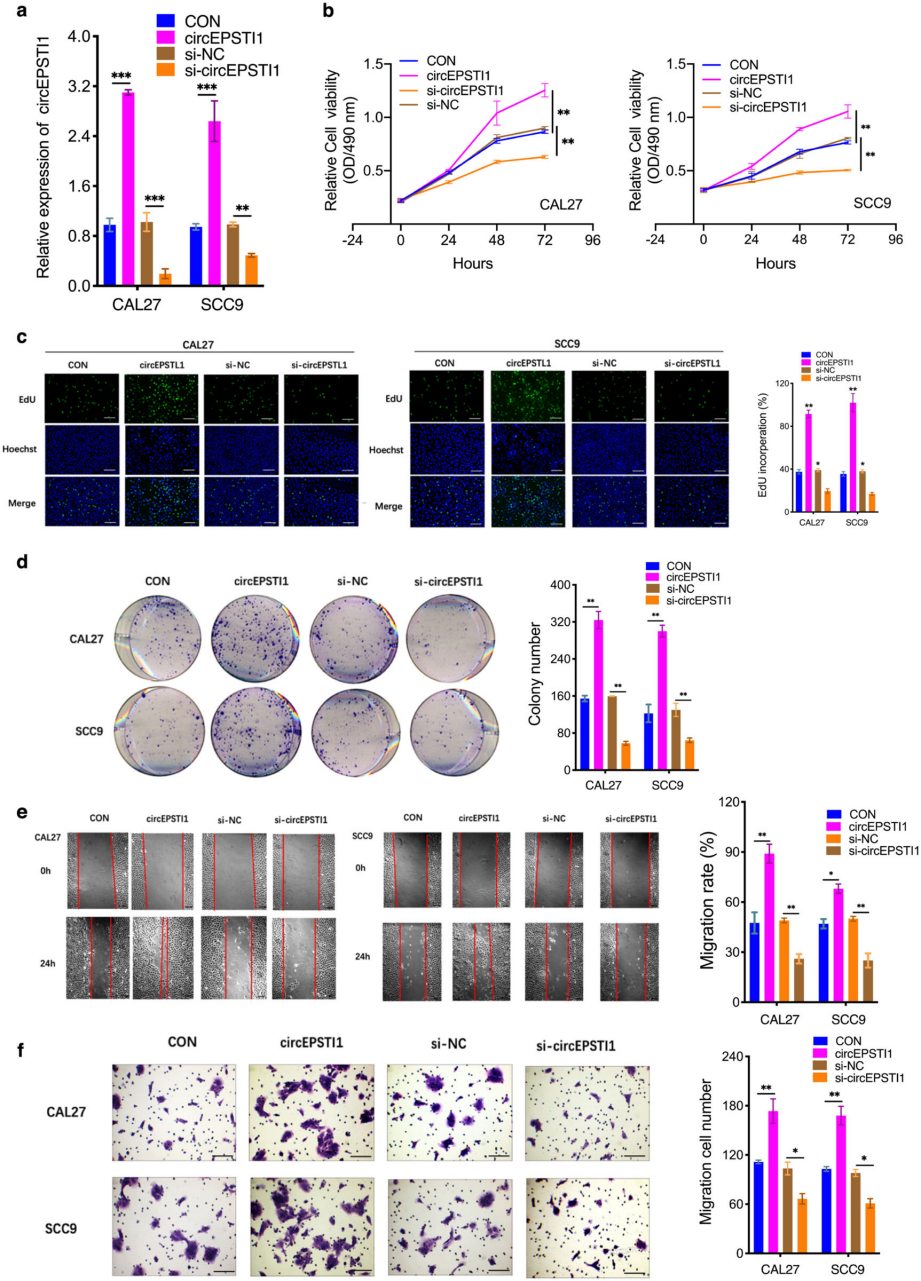
CircEPSTI1 overexpression promotes malignant behaviors of OSCC cells. **a** The level of circEPSTI1 was increased or decreased after transfection with an overexpression vector or siRNA against circEPSTI1, respectively. **b**–**d** CircEPSTI1 promoted the proliferation of OSCC cells, as shown by CCK8 (**b**), EdU (**c**, scale bar 100 μm), and colony formation (**d**) assays. **e**, **f** The migration and invasion abilities of OSCC cells were assessed by wound healing (**e** scale bar 500 μm) and transwell (**f** scale bar 100 μm) assays. ***P < 0.001; **P < 0.01; *P < 0.05.

To investigate the potential functions of circEPSTI1 in regulating OSCC cell biological behaviors, we first designed two si-circEPSTI1 constructs to target the back splice sequence of circEPSTI1. RT-qPCR in CAL27 and SCC9 cells showed that si-circEPSTI1-2 had a higher circEPSTI1 downregulation efficiency and was selected for further RNA analysis (Supplemental Fig. Sb). Moreover, neither si-circEPSTI1 construct affected the expression of linear EPSTI1 species (Supplemental Fig. Sc). Via transfection with overexpression or siRNA vectors targeting circEPSTI1, circEPSTI1 was overexpressed or knocked down, respectively, in CAL27 and SCC9 cells (Fig. 2a). Growth curve assays demonstrated that circEPSTI1 upregulation significantly enhanced the proliferation and viability of CAL27 and SCC9 cells, whereas circEPSTI1 downregulation inhibited cell growth (Fig. 2b). Similarly, the results of EdU assays revealed that circEPSTI1 overexpression markedly increased the number of EdU-positive cells, while circEPSTI1 knockdown produced the opposite effect (Fig. 2c). The results of colony formation assays further demonstrated that the colony-forming ability of CAL27 and SCC9 cells was significantly enhanced by circEPSTI1 upregulation and impaired by circEPSTI1 downregulation (Fig. 2d). The results of wound healing (Fig. 2e) and transwell (Fig. 2f) assays indicated that the migration and invasion abilities of CAL27 and SCC9 cells were markedly increased by circEPSTI1 upregulation but significantly suppressed by circEPSTI1 downregulation. Cell-cycle analysis revealed that knockdown of circEPSTI1 led to increased percentages of CAL27 cells in G0-G1 phases and decreased percentages in S phase, suggesting that downregulation of circEPSTI1 resulted in G1 arrest in OSCC cells (Supplemental Fig. Sd). These results indicated that circEPSTI1 can promote OSCC growth.

### CircEPSTI1 functions as a sponge for miR-942-5p to accelerate epithelial-mesenchymal transition (EMT)

**Fig. 3:**
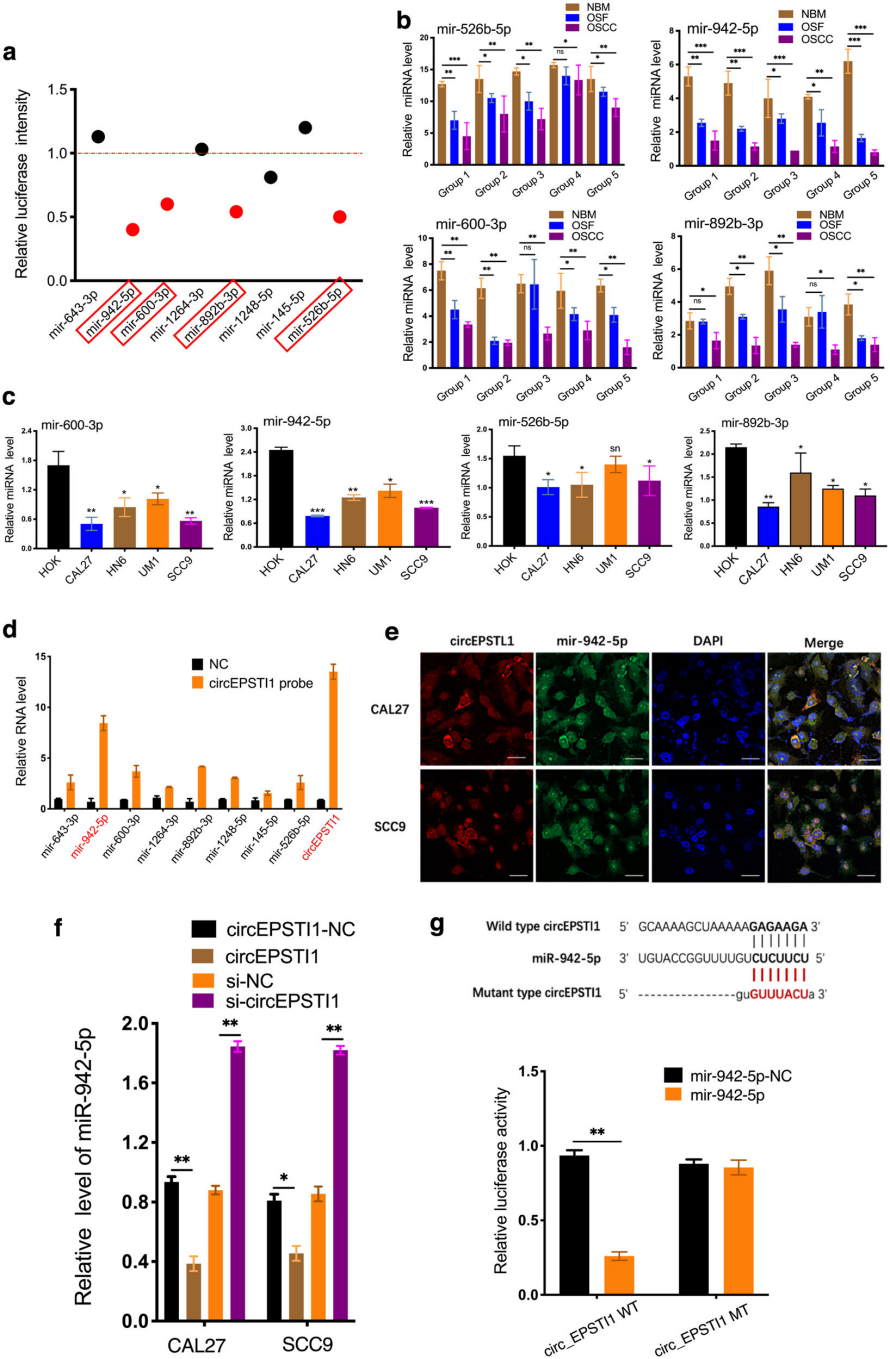
CircEPSTI1 functions as a sponge for miR-942-5p to accelerate EMT. **a** The luciferase intensity was reduced by more than 40% in response to transfection of miR-942-5p, miR-600-3p, miR-892b-3p, and miR-526b-5p, as determined by luciferase activity assays. **b**, **c** Four candidate miRNAs in tissue samples (**b**) and OSCC cell lines (**c**). **d** In RIP pulldown experiments, miR-942-5p showed the greatest enrichment in the pulldown with a specific circEPSTI1 probe compared to that with the negative control probe. **e** The subcellular localization of both circEPSTI1 (red) and miR-942-5p (green) was cytoplasmic, as determined by FISH (scale bar 10 μm). **f** Overexpression or silencing of circEPSTI1 markedly decreased or increased the expression of miR-942-5p, respectively. **g** The dual luciferase reporter assay results indicated that miR-942-5p mimics significantly decreased the luciferase activity of the circEPSTI1-WT construct. **i**. ***P < 0.001; **P < 0.01; *P < 0.05.

The top eight potential interacting miRNAs (miR-942-5p, miR-600-3p, miR-1248-5p, miR-1264-3p, miR-643-3p, miR-145-5p, miR-892b-3p, and miR-526b-5p) with circEPSTI1 (Supplemental Fig. Se) were selected from the Circular RNA Interactome database (https://circinteractome.nia.nih.gov/) in accordance with the positions of their putative binding sites in the 3′-untranslated region (3′UTR) of the circEPSTI1 sequence. Subsequently, luciferase reporter assays were used to determine whether these miRNAs can directly target the 3′UTR of circEPSTI1. HEK 293 T cells were cotransfected with each miRNA mimic and luciferase reporter for luciferase activity assays. The luciferase intensity was reduced by more than 40% in response to transfection with miR-942-5p, miR-600-3p, miR-892b-3p, and miR-526b-5p (Fig. 3a). However, co-transfection of miRNA mimics and the mutated luciferase reporter did not significantly affect luciferase activity (Supplemental Fig. Sf). Furthermore, miR-942-5p, miR-600-3p, miR-892b-3p, and miR-526b-5p were also selected for RT-qPCR validation in tissue samples and OSCC cell lines. The expression of miR-942-5p was found to decrease most significantly among these miRNAs in both tissue specimens and cell lines (Fig. 3b, c). Moreover, the expression of miR-942-5p was higher in si-circEPSTI1-transfected cells than in control cells (Supplemental Fig. Sg). The results of RIP pulldown experiments showed that miR-942-5p exhibited the greatest enrichment in the pulldown with a specific circEPSTI1 probe compared to that with the negative control probe, indicating that miR-942-5p could be a critical circEPSTI1-associated miRNA in OSCC cells (Fig. 3d). Hence, miR-942-5p was considered the candidate target miRNA of circEPSTI1 in this study.

CircRNAs can act as miRNA sponges in the cytoplasm, and we found by a FISH assay that circEPSTI1 (red) and miR-942-5p (green) were localized primarily in the cytoplasm of CAL27 and SCC9 cells (Fig. 3e). Overexpression or silencing of circEPSTI1 markedly decreased or increased miR-942-5p expression, respectively (Fig. 3f). Moreover, transfection with miR-942-5p mimics significantly decreased the luciferase activity of the circEPSTI1-WT construct but not the mutant construct (Fig. 3g), suggesting that circEPSTI1 and miR-942-5p might interact directly. MiRNAs regulate the expression of their target genes by binding to Argonaute 2 (AGO2), the key component of the RNA-induced silencing complex (RISC). Thus, an anti-AGO2 RIP assay was conducted in CAL27 cells to pulldown the RNA transcripts that bind to AGO2 with an anti-AGO2 antibody, and IgG was used as the negative control. AGO2, circEPSTI1, and miR-942-5p were efficiently pulled down by the anti-AGO2 antibody. Both circEPSTI1 and miR-942-5p were significantly enriched in cells transfected with miR-942-5p mimics (Supplemental Fig. Sh). Moreover, circEPSTI1 and miR-942-5p but not circANRIL (a circRNA that reportedly does not bind to AGO2), were significantly enriched, as they were precipitated by the anti-AGO2 antibody (Supplemental Fig. Sh).

**Fig. 4:**
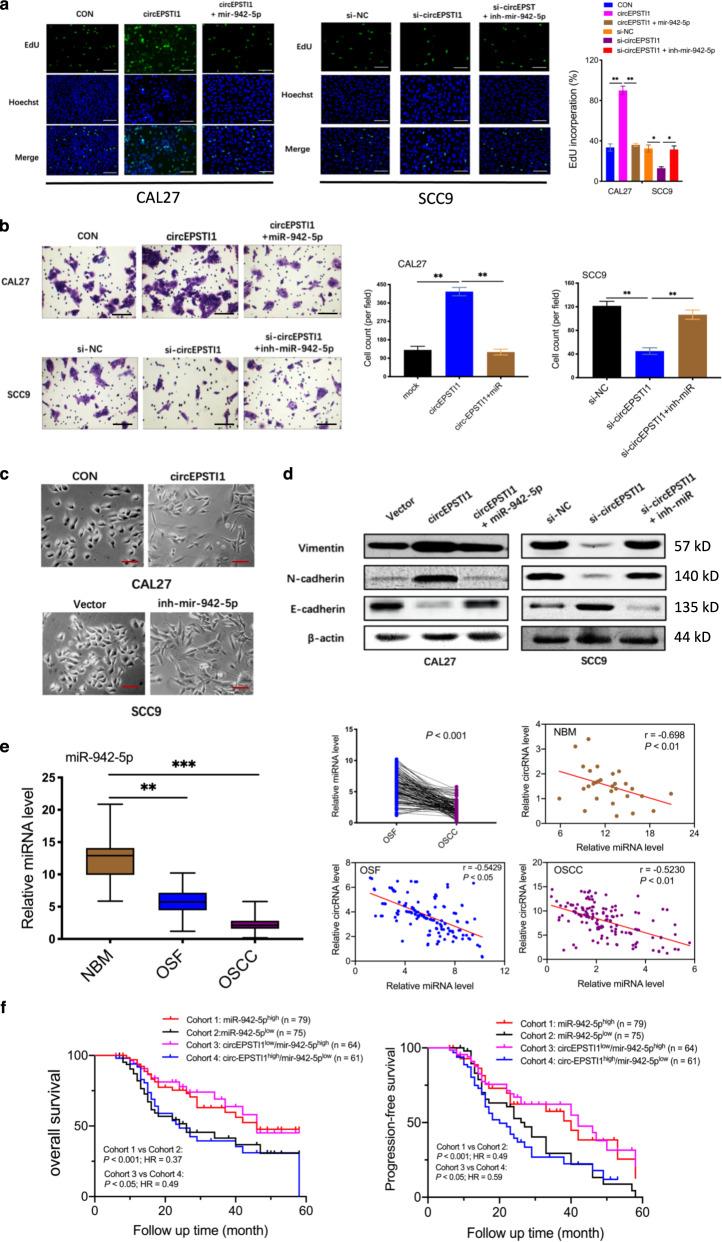
CircEPSTI1 functions as a sponge for miR-942-5p to accelerate EMT. **a** The results of rescue experiments indicated that miR-942-5p reversed the promotive or suppressive effects of circEPSTI1 overexpression or knockdown, respectively, on proliferation and invasion, as demonstrated by an EdU assay (scale bar 100 μm)**. b** Rescue experiments indicated that miR-942-5p reversed the promotive or suppressive effects of circEPSTI1 overexpression or knockdown, respectively, on proliferation and invasion, as demonstrated by transwell assays (scale bar 100 μm). **c** OSCC cells with circEPSTI1 overexpression and miR-942-5p knockdown displayed a spindle-like morphology (scale bar 50 μm). **d** The western blot analysis results revealed that the expression of EMT molecules induced by circEPSTI1 overexpression or knockdown was rescued by miR-942-5p mimics or inhibitors, respectively. **e** The expression of miR-942-5p in 154 OSCC and matched OSF tissues was analyzed by RT-qPCR. **f** Kaplan–Meier analysis of the correlation between the expression of miR-942-5p or circEPSTI1/miR-942-5p and OS as well as PFS. ***P < 0.001; **P < 0.01; *P < 0.05.

To explore whether circEPSTI1 biologically functions as a sponge for miR-942-5p, rescue experiments using miR-942-5p mimics and inhibitors were designed. MiR-942-5p mimics reversed the circEPSTI1-induced proliferation and invasion of CAL27 OSCC cells, whereas miR-942-5p inhibitors rescued the suppressive effects of circEPSTI1 knockdown in SCC9 cells, as evidenced by the results of EdU (Fig. 4a) and transwell (Fig. 4b) assays. As EMT can enhance progression and invasion in various cancers, we investigated whether the circEPSTI1/miR-942-5p axis promotes EMT. As expected, CAL27 cells with circEPSTI1 overexpression and SCC9 cells with miR-942-5p knockdown acquired a spindle-like morphology, with sharp edges compared with those of vector-transfected cells (Fig. 4c). Moreover, western blot analysis revealed that regardless of whether circEPSTI1 was overexpressed in CAL27 cells or knocked down in SCC9 cells, the expression level of E-cadherin was decreased or increased when the expression levels of N-cadherin and Vimentin were increased or decreased, respectively. the changes in the expression of E-cadherin, N-cadherin, and Vimentin induced by overexpression and knockdown of circEPSTI1 were reversed by miR-942-5p mimics and inhibitors, respectively (Fig. 4d). These results suggest that circEPSTI1 contributes to the promotion of EMT by sponging miR-942-5p in OSCC cells.

The expression level of miR-942-5p was measured in 154 pairs of OSCC tissues in the background of OSF and in 30 unmatched NBM tissues. MiR-942-5p was markedly and consistently downregulated sequentially from NBM to OSF to OSCC tissues (Fig. 4e). Pearson correlation analysis displayed a significant negative correlation between the expression of circEPSTI1 and that of miR-942-5p in NBM, OSF, and OSCC tissues (Fig. 4e). As shown in Table 1, low expression of miR-942-5p was significantly correlated with high T stage (*P* < 0.01) and advanced TNM stage (*P* < 0.01). Kaplan–Meier survival analysis (Fig. 4f) showed that a lower level of miR-942-5p expression was correlated with poorer OS and PFS. Moreover, OSCC patients with both circEPSTI1^high^ and miR-942-5p^low^ expression (denoted circEPSTI1^high^/miR-942-5p^low^) had poorer OS and PFS than patients with the circEPSTI1^low^/miR-942-5p^high^ expression profile. Hence, either miR-942-5p expression alone or circEPSTI1/miR-942-5p expression combined could be a prognostic biomarker for OSCC in the background of OSF.

### CircEPSTI1 promotes OSCC cell proliferation and invasion through the circEPSTI1/miR-942-5p/LTBP2 axis

**Fig. 5:**
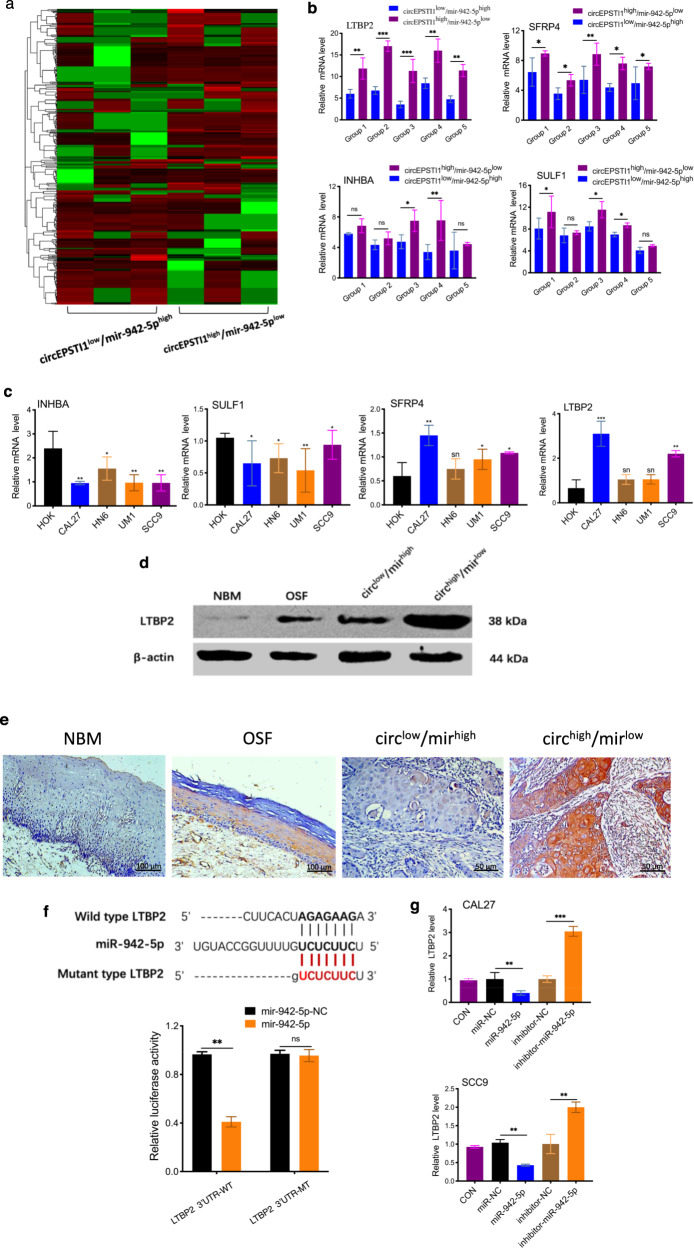
circEPSTI1 promotes OSCC cell proliferation and invasion through the circEPSTI1/miR-942-5p/LTBP2 axis. **a** Heat map of high-density oligonucleotide microarray analysis results for three circEPSTI1^high^/miR-942-5p^low^ OSCC tissues and three circEPSTI1^low^/miR-942-5p^high^ OSCC tissues. **b** The expression levels of the four top potential target mRNAs in circEPSTI1^high^/miR-942-5p^low^ OSCC tissues were compared to those in circEPSTI1^low^/miR-942-5p^high^ OSCC tissues. **c** The mRNA transcript level of LTBP2 was the most significantly increased in cell lines, as determined by RT-qPCR. **d** The protein expression level of LTBP2 was highest in circEPSTI1^high^/miR-942-5p^low^ OSCC tissue, followed sequentially by circEPSTI1^low^/miR-942-5p^high^ OSCC tissue, OSF tissue and NBM tissue. **e** Characteristic IHC staining of LTBP2 in circEPSTI1^high^/miR-942-5p^low^ OSCC tissue, circEPSTI1^low^/miR-942-5p^high^ OSCC tissue, matched OSF tissue, and NBM tissue. **f** The activity of the luciferase reporter vector carrying the LTBP2 3’UTR-WT sequence was significantly decreased by miR-942-5p mimics, as demonstrated by a dual luciferase reporter assay. **g** Mir-942-5p mimics or inhibitors obviously reduced or enhanced the expression of LTBP2, respectively. ***P < 0.001; **P < 0.01; *P < 0.05.

To identify differentially expressed genes and pathways that could result in differences in prognosis between patients with circEPSTI1^high^/miR-942-5p^low^ OSCC and those with circEPSTI1^low^/miR-942-5p^high^ OSCC, we performed high-density oligonucleotide microarray analysis of three circEPSTI1^high^/miR-942-5p^low^ OSCCs and three circEPSTI1^low^/miR-942-5p^high^ OSCCs (Fig. 5a). A total of 533 differentially altered genes were identified (Supplemental Materials S2) based on the expression pattern of genes with a change of either 2-fold change (induction) or 0.5-fold (repression). Eventually, 449 upregulated and 84 downregulated probe sets with significantly different expression levels were selected by significance analysis of microarrays (SAM). However, among the top 20 differentially expressed genes, only seven (indicated by the green gene symbols in Supplemental Materials S2) were identified as potential target genes of miR-942-5p in the TargetScan 7.1 database (http://www.targetscan.org). We selected the four top potential target genes (INHBA, SFRP4, LTBP2, and SULF1) for our further experiments. All genes were upregulated significantly in 5 circEPSTI1^high^/miR-942-5p^low^ OSCC tissues compared to five circEPSTI1^low^/miR-942-5p^high^ OSCC tissues (Fig. 5b). The RT-qPCR results indicated that the mRNA transcript levels of only LTBP2 and SFRP4 were significantly increased in the cell lines; however, LTBP2 exhibited stronger upregulation than SFRP4 (Fig. 5c). Thus, we selected LTBP2 as the candidate gene for further analysis. The western blot analysis results showed that LTBP2 expression was highest in circEPSTI1^high^/miR-942-5p^low^ OSCC tissue, followed sequentially by circEPSTI1^low^/miR-942-5p^high^ OSCC tissue, OSF tissue, and NBM tissue (Fig. 5d). Moreover, the characteristic staining of LTBP2 in circEPSTI1^high^/miR-942-5p^low^ OSCC tissue, circEPSTI1^low^/miR-942-5p^high^ OSCC tissue, OSF tissue, and unmatched NBM tissue identified by IHC analysis is shown in Fig. 5e.

**Fig. 6:**
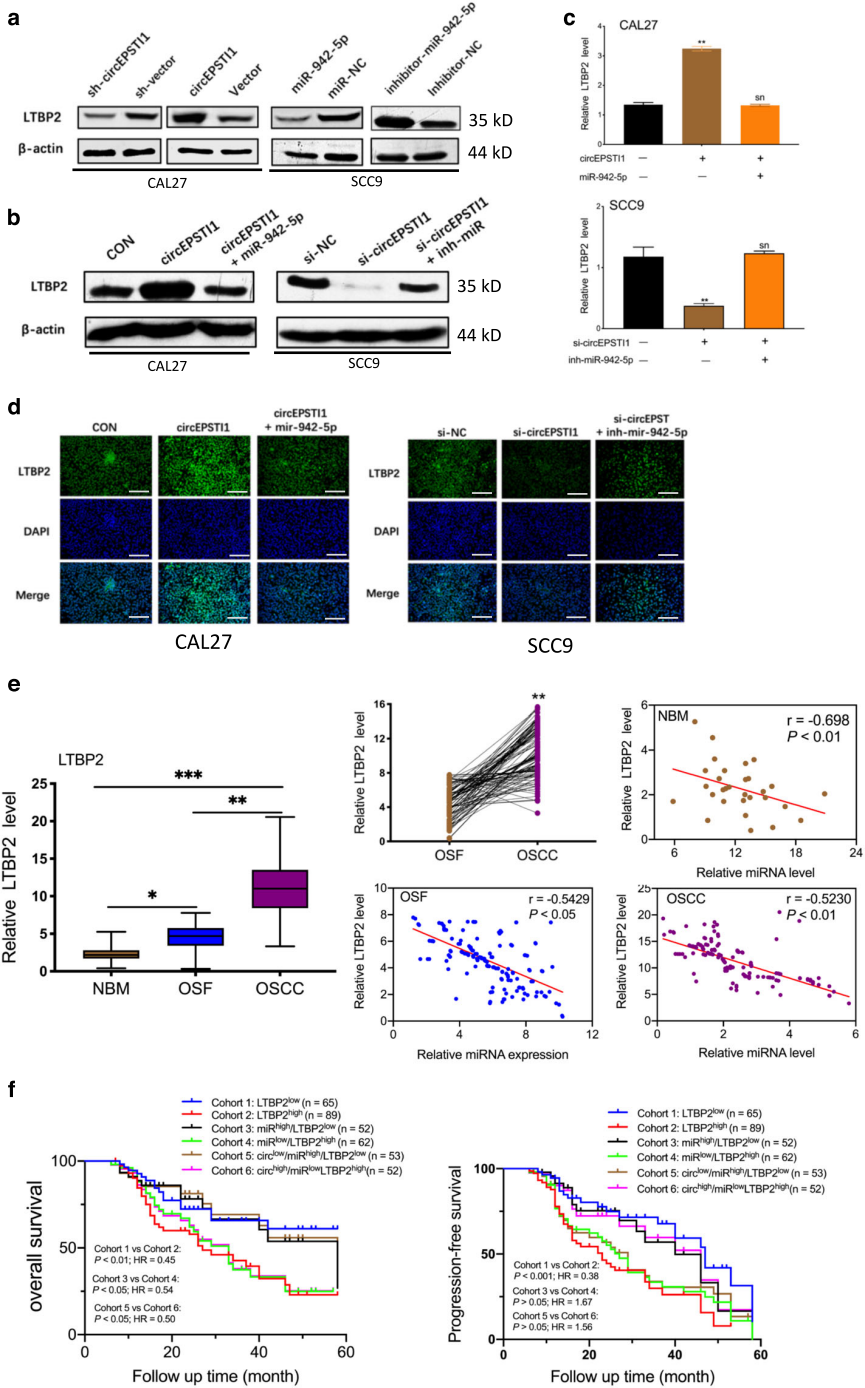
circEPSTI1 promotes OSCC cell proliferation and invasion through the circEPSTI1/miR-942-5p/LTBP2 axis. **a** The protein levels of LTBP2 were accordingly altered by treatment with circEPSTI1 or miR-942-5p. **b**, **c** Knockdown or overexpression of circEPSTI1 markedly decreased or increased the expression of LTBP2 at both the protein (**b**) and mRNA (**c**) levels, which can be reversed by miR-942-5p mimics or inhibitors. **d** The increase or decrease in the expression level of LTBP2 induced by circEPSTI1 overexpression or knockdown was reversed by miR-942-5p mimics or inhibitors as demonstrated by IF staining (scale bar 100 μm). **e** The expression level of LTBP2 in 154 OSCC and matched OSF tissues was analyzed by real-time PCR. **f** Kaplan–Meier analysis of the correlation between the expression levels of LTBP2, circEPSTI1/LTBP2, miR-942-5p/LTBP2, or circEPSTI1/miR-942-5p/LTBP2 and OS as well as PFS. ***P < 0.001; **P < 0.01; *P < 0.05.

The dual luciferase reporter assay results showed that the activity of the luciferase reporter vector carrying the LTBP2 3’UTR-WT sequence was significantly decreased by miR-942-5p mimics (Fig. 5f). Moreover, in CAL27 and SCC9 cells, miR-942-5p mimics obviously reduced but miR-942-5p inhibitors significantly enhanced the level of LTBP2 (Fig. 5g). The protein expression levels of LTBP2 were accordingly altered in OSCC cells (Fig. 6a). Based on these data, we concluded that LTBP2 is directly targeted by miR-942-5p.

Furthermore, as shown in Fig. 6a, b, knockdown or overexpression of circEPSTI1 markedly decreased or increased the expression of LTBP2, respectively, at both the mRNA and protein levels. However, the increase or decrease in LTBP2 expression correspondingly induced by circEPSTI1 overexpression or knockdown was reversed by miR-942-5p mimics or inhibitors, respectively, at both the mRNA (Fig. 6c) and protein levels (Fig. 6b). Immunofluorescence (IF) staining showed that overexpression of circEPSTI1 significantly increased the expression of LTBP2 and that this effect was abolished by miR-942-5p mimics; in contrast, downregulation of circEPSTI1 reduced LTBP2 expression, and this effect was counteracted by miR-942-5p inhibitors (Fig. 6d). These data suggest that circEPSTI1 can regulate the expression of LTBP2 by serving as a competing endogenous RNA (ceRNA) for miR-942-5p in OSCC.

In addition, the expression level of LTBP2 was measured in 154 pairs of tissues from OSCC patient with OSF and in 30 unmatched NBM tissues. LTBP2 was markedly and consistently upregulated sequentially from NBM to OSF to OSCC tissues (Fig. 6e). As indicated in Table 1, high expression of LTBP2 was significantly correlated with high T stage (*P* < 0.01) and advanced TNM stage (*P* < 0.01). Kaplan–Meier survival analysis showed that patients with high LTBP2 expression had poorer OS and PFS (Fig. 6f). Furthermore, patients with miR-942-5p^low^/LTBP2^high^ or circEPSTI1^high^/miR-942-5p^low^/LTBP2^high^ expression profiles had poorer OS but not poorer PFS than patients with miR-942-5p^high^/LTBP2^low^ or circEPSTI1^low^/miR-942-5p^high^/LTBP2^low^ expression profiles (Fig. 6f, and Table 2). Hence, the expression level of LTBP2 alone or circEPSTI1/miR-942-5p/LTBP2 combined could serve as a prognostic biomarker for OSCC in the background of OSF.

### CircEPSTI1 promotes OSCC progression through the phosphorylation of PI3K/Akt/mTOR signaling pathway components

**Fig. 7:**
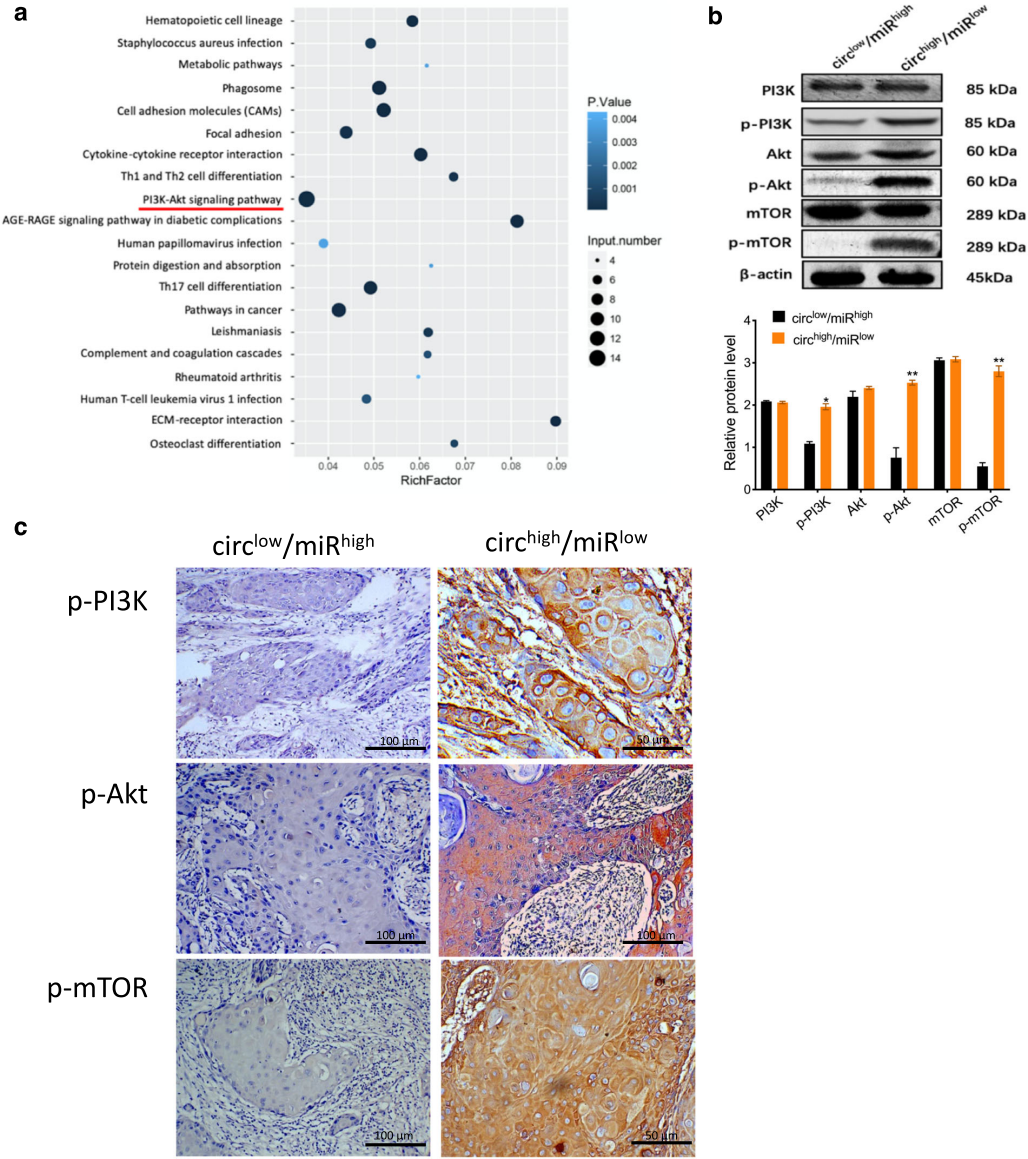
CircEPSTI1 promotes the progression of OSCC through the phosphorylation of PI3K/Akt/mTOR signaling pathway components. **a** KEGG analysis of all differentially altered genes between circEPSTI1^high^/miR-942-5p^low^ OSCCs and circEPSTI1^low^/miR-942-5p^high^ OSCCs. **b** The significantly increased levels of phosphorylated PI3K, Akt, and mTOR proteins in circEPSTI1^high^/miR-942-5p^low^ OSCC tissues. **c** Characteristic IHC staining of p-PI3K, p-Akt, and p-mTOR in circEPSTI1^high^/miR-942-5p^low^ and circEPSTI1^low^/miR-942-5p^high^ OSCC tissues. ***P < 0.001; **P < 0.01; *P < 0.05.

Among the 204 signaling pathways identified by KEGG pathway analysis as differentially enriched between circEPSTI1^high^/miR-942-5p^low^ OSCCs and circEPSTI1^low^/miR-942-5p^high^ OSCC tissues, the PI3K/Akt/mTOR pathway contained the most differentially expressed genes and exhibited the minimum *P* value (Fig. 7a). In circEPSTI1^high^/miR-942-5p^low^ OSCC tissues, activation of the PI3K/Akt/mTOR pathway was indicated by the significantly increased levels of phosphorylated PI3K (p-PI3K), Akt (p-AKt), and mTOR (p-mTOR) proteins compared to those in circEPSTI1^low^/miR-942-5p^high^ OSCC tissues, although the levels of total PI3K, Akt, and mTOR did not differ (Fig. 7b). Images showing characteristic IHC staining of p-PI3K, p-Akt, and p-mTOR in circEPSTI1^high^/miR-942-5p^low^ OSCC and circEPSTI1^low^/miR-942-5p^high^ OSCC tissues are presented in Fig. 7c.

**Fig. 8:**
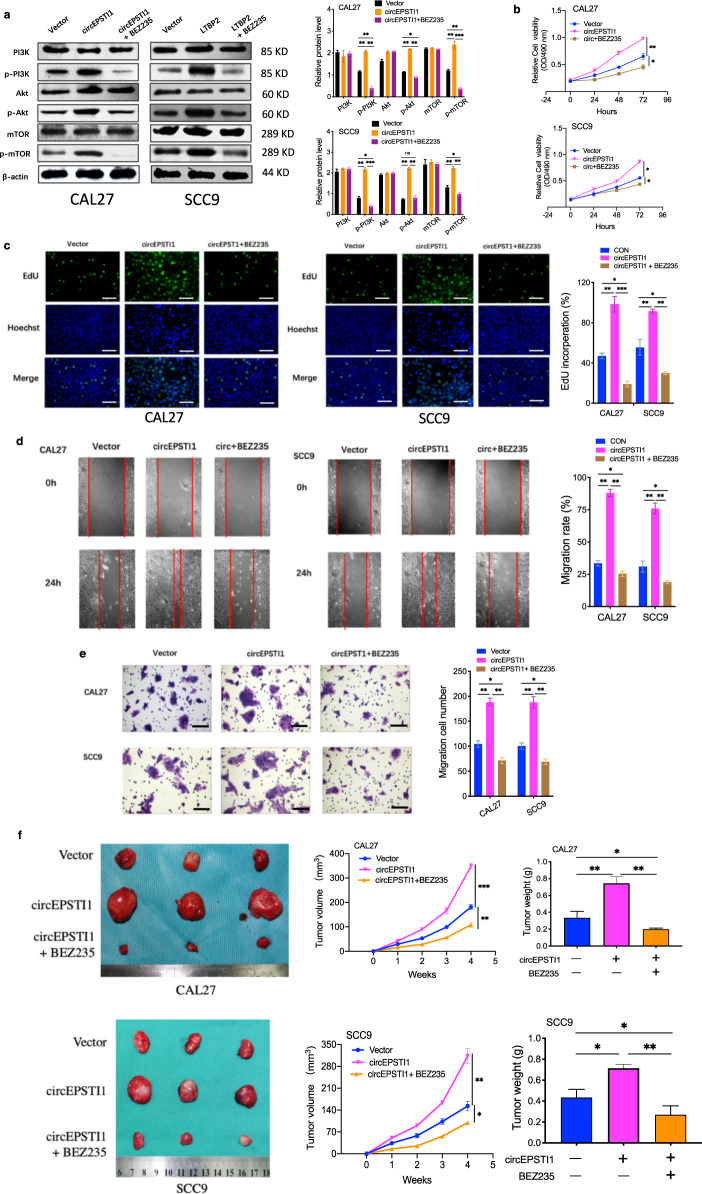
BEZ235 attenuates the circEPSTI1 overexpression-induced proliferation and invasion of OSCC cells. **a** The increases in the protein levels of p-PI3K, p-Akt, and p-mTOR after circEPSTI1 overexpression were significantly attenuated by BEZ235 treatment. **b**–**f** BEZ235 attenuated the circEPSTI1 overexpression-induced proliferation and invasion of OSCC cells, as evidenced by the growth curve assays (**b**), EdU assays (**c**, scale bar 100 μm), wound healing assays (**d** scale bar 500 μm), and transwell assays (**e** scale bar 100 μm) and nude mouse xenograft assays in vivo (**f**). ***P < 0.001; **P < 0.01; *P < 0.05.

Furthermore, BEZ235, a PI3k/mTOR dual inhibitor, was added to the medium of CAL27 and SCC9 cells with overexpression of circEPSTI1, and the increases in the protein levels of p-PI3K, p-Akt, and p-mTOR induced by circEPSTI1 overexpression were significantly attenuated (Fig. 8a). The results of the growth curve (Fig. 8b), EdU (Fig. 8c), wound healing (Fig. 8d), and transwell (Fig. 8e) assays also indicated that the PI3k/mTOR inhibitor attenuates OSCC progression caused by circEPSTI1 overexpression. To determine the effects of BEZ235 on tumor growth in vivo, CAL27 and SCC9 cells were stably transfected with vector and circEPSTI1 and were then subcutaneously injected into nude mice, which were treated or not treated with BEZ235. BEZ235 treatment significantly reduced the size and weight of tumors derived from cells overexpressing circEPSTI1 and controls (Fig. 8f). Collectively, these results indicate that the circEPSTI1/miR-942-5p/LTBP2 axis promotes OSCC development through the phosphorylation of PI3K/Akt/mTOR signaling pathway components.

## Discussion

OSCC in the background of OSF caused by areca nut chewing has a high incidence in some provinces of China and in Asia-Pacific countries due to the lack of effective diagnostic biomarkers. CircRNAs have recently become widely used cancer biomarkers, but little is known about the role of circRNA biomarkers in OSCC with OSF. Numerous biomarkers differentially expressed at both the mRNA and protein levels between normal oral mucosa and OSCC or OSF tissues have been identified^20–22^. To our knowledge, our present study is the first comprehensive study on the expression profiles and regulatory functions of circRNAs in OSCC with OSF. Via high-throughput RNA-seq, we identified that circEPSTI1 was sequentially upregulated from NBM to OSF to OSCC. CircEPSTI1 is derived from the EPSTI1 gene, which is characterized by its participation in extensive epithelial-stromal interactions, innate immunity, and cancer invasion and metastasis^23,24^. Guo et al. assessed and identified circEPSTI1 as an outstanding potential diagnostic biomarker for systemic lupus erythematosus (SLE)^25^. Moreover, circEPSTI1 was found to regulate ovarian cancer progression and could be used as a biomarker and therapeutic target in ovarian cancer^26^. In our present study, we demonstrated for the first time that circEPSTI1 expression is increased in OSCC and that overexpressing or silencing circEPSTI1 can respectively promote or inhibit OSCC cell proliferation and invasion. Additionally, high expression of circEPSTI1 in OSCC patients predicted poor prognosis. Therefore, circEPSTI1 could serve as a prognostic biomarker for OSCC in the background of OSF.

Many studies have revealed that circRNAs control oncogenes or tumor suppressors mainly via a circRNA-miRNA-mRNA axis^27,28^. Via bioinformatics methods combined with RNA pulldown assays, we revealed that circEPSTI1 exerts its regulatory effects by binding miR-942-5p to upregulate the expression of LTBP2, which is also directly targeted by miR-942-5p. Studies have reported that miR-942-5p can regulate the progression and metastasis of various cancers, but research on the role of miR-942-5p in the regulation of OSCC progression is scarce^26^. EMT is a common phenomenon in epithelial-derived malignant tumors and is characterized by loss of cell–cell junctions and acquisition of a spindle morphology to establish a motile and invasive phenotype^29^. Hallmarks of EMT include loss of expression and function of epithelial markers such as E-cadherin, cytokeratin, and claudin, as well as concomitant increases in the abundances of mesenchymal markers, such as vimentin and N-cadherin. The present study investigated whether the circEPSTI1/miR-942-5p axis affects EMT in OSCC cells. The results provided the first evidence that miR-942-5p is downregulated in OSCC and can reverse the deregulated expression of epithelial markers and mesenchymal markers in the setting of EMT caused by circEPSTI1 upregulation or downregulation, suggesting that circEPSTI1 contributes to EMT by sponging miR-942-5p in OSCC.

In our present study, the fold change in the expression of LTBP2, which is targeted by miR-942-5p, was obviously higher in circEPSTI1^high^/miR-942-5p^low^ OSCC tissues than in circEPSTI1^low^/miR-942-5p^high^ OSCC tissues. LTBP2 belongs to the LTBP/fibrillin family of extracellular matrix (ECM) proteins and performs an important function in modulating the structural integrity of the ECM^30^. Various functions of LTBP2 have been indicated in different types of cancer. Increased expression of LTBP2 has been observed in head and neck squamous cell carcinoma^31^, thyroid cancer^32^, and liver cancer^33^ and was associated with unfavorable outcomes and tumor progression. In contrast, LTBP2 is downregulated and performs a tumor-suppressive function in esophageal squamous cell carcinoma and nasopharyngeal carcinoma^34,35^. These findings suggest that LTBP2 performs either an oncogenic or a tumor-suppressive function in a cell type- or context-dependent manner. However, the expression pattern of LTBP2 in OSCC and OSF remains unknown. In the present study, we found for the first time that LTBP2 is consistently upregulated sequentially from NBM to OSF to OSCC and that its expression is affected by circEPSTI1 and miR-942-5p, indicating that the circEPSTI1/miR-942-5p/LTBP2 axis can promote OSCC progression.

For the first time, we identified the PI3K/Akt signaling pathway as the most altered pathway between the circEPSTI1^high^/miR-942-5p^low^ and circEPSTI1^low^/miR-942-5p^high^ profiles. The PI3K/Akt signaling pathway regulates cell proliferation, invasion, and apoptosis and, when deregulated, promotes tumorigenesis^36^.

Therefore, targeting the PI3K/Akt pathway may constitute an attractive therapeutic approach for various cancers. The dual PI3k/mTOR inhibitor BEZ235 has been approved by the FDA for the treatment of various cancers. BEZ235 inhibits the activity of multiple class I PI3K isoforms and mTORC1/2 kinase, exerting potent anticancer activity and attenuating PI3k reactivation and mTORC2-mediated Akt reactivation. BEZ235 can thus prevent feedback reactivation of Akt due to its dual blockade effects upstream and downstream of Akt^37^. We found that BEZ235 reduces OSCC cell proliferation and invasion caused by overexpression of circEPSTI1 or LTBP2 in vitro and in vivo. These data suggest that targeting the PI3K/Akt/mTOR pathway, especially with a dual PI3K/mTOR inhibitor, may efficiently treat OSCC that develops in the background of OSF.

In summary, we found that circEPSTI1 expression is elevated in OSCC and OSF, both of which are prevalent lesions in China and in South/Southeast Asian countries and regions. In addition, our evidence indicated that circEPSTI1/miR-942-5p/LTBP2 axis can promote the proliferation, migration, and invasion of OSCC cells in vitro and in vivo via activation of EMT and PI3K/Akt/mTOR signaling pathway. Thus, circEPSTI1 could be an effective prognostic biomarker for OSCC in the background of OSF.
